# Iodine consumption and cognitive performance: Confirmation of adequate consumption

**DOI:** 10.1002/fsn3.694

**Published:** 2018-06-01

**Authors:** Hani Choudhry, Md. Nasrullah

**Affiliations:** ^1^ Department of Biochemistry Faculty of Science King Abdulaziz University Jeddah Saudi Arabia; ^2^ Cancer and Mutagenesis Unit King Fahd Center for Medical Research King Abdulaziz University Jeddah Saudi Arabia

**Keywords:** cognitive performance, cretinism, dietary plan, food salt iodization program, iodine

## Abstract

Iodine, a dynamic nutrient present in thyroid hormones, is responsible for regulating thyroid function, supporting a healthy metabolism, and aiding growth and development. Iodine is also essential for brain development during specific time windows influencing neurogenesis, neuronal and glial cell differentiation, myelination, neuronal migration, and synaptogenesis. About 1.5 billion people in 130 countries live in areas at risk of iron deficiencies (IDs). Reduced mental ability due to IDs occurs in almost 300 million people. Ensuring the consumption of minimum recommended daily allowances of iodine remains challenging. The effects of ID disorders range from high mortality of fetuses and children to inhibited mental development (cretinism). Poor socioeconomic development and impaired school performance are also notable. Currently, ID disorders are the single greatest contributor to preventable brain damage in fetuses and infants and arrested psychomotor development in children. Iodized salt may help fulfill iodine requirements. Increases in food salt iodization programs can help overcome ID disorders. Dietary plans can be well adjusted to incorporate iodinated foods. Maternal iodine supplementation for offspring requires adequate attention. Fruits, vegetables, bread, eggs, legumes (beans and peas), nuts, seeds, seafood, lean meats and poultry, and soy products provide small quantities of iodine. Nutrient‐dense foods containing essential vitamins and minerals such as iodine may confer positive effects. To some extent, fortified foods and daily dietary supplements can be provided for different nutrients including iodine; otherwise, iodine may be consumed in less than the recommended amounts. This review focuses on aspects of adequate iodine consumption to avoid cognitive impairments.

## 1 INTRODUCTION

The thyroid hormones (THs) 3,5,3′‐triiodo‐L‐thyronine (T3) and 3,5,3′,5′‐tetraiodo‐L‐thyronine (T4) are important for brain development and proper brain function throughout life and iodine is important for the production of these hormones (Abel et al., 2017; Schroeder & Privalsky, 2014). Almost 1.5 billion people in 130 countries are estimated to be at risk for iodine deficiency (ID; World Health Organization, 2006). The consequences of ID are important (Delange, 1994). Rather than causing a single disease, ID causes many disturbances in the body, which are termed ID disorders (IDDs; Ahad & Ganie, 2010; Eastman & Zimmermann, 2000; Kapil, 2007). They include a spectrum of different disorders such as hypothyroidism, stillbirths, goiters, congenital abnormalities, and impaired growth and development in children (Chen & Hetzel, 2010; Kapil & Sareen, 2012). Additionally, ID increases infant mortality and is the leading preventable cause of mental deficiency in childhood, although many other factors are influential (Nyaradi, Li, Hickling, Foster, & Oddy, 2013; West, n.d.). Epidemiological studies have demonstrated the relationship between ID and cretinism (Bailey, West, & Black, 2015). IDDs constitute the single greatest reason of preventable brain damage in fetuses and infants and also of retarded psychomotor development in young children (Chevrier et al., 2011; Costeira et al., 2011). At least 1.2 million children each year are born with cretins and are mentally retarded, physically stunted, deaf–mute, or paralyzed due to the outcome of ID (“Assessment of iodine deficiency disorders and monitoring their elimination: A guide for programme managers, 2007; Indicators for assessing iodine deficiency disorders and their control through salt iodization, 1994). In addition, an estimated yearly total of at least 60,000 miscarriages, stillbirths, and neonatal deaths stem from severe ID in early pregnancy (Zupan & Åhman, 2006). The prevalence of severe ID has reduced recently and the problems of ID re‐emerged in diverse vulnerable populations, such as in infants and pregnant women. By correcting IDs before pregnancy, all these conditions can be majorly prevented (Rohner et al., 2014).

Various approaches remain that aid in overcoming ID. Among them, the universal salt iodization (USI) program is considered as the main intervention (Nouri Saeidlou, Babaei, Ayremlou, & Entezarmahdi, 2017; Prevention of Micronutrient Deficiencies, 1998). Salt has been chosen as the product that can be fortified for several reasons; it is manufactured centrally or in few industries, the expense of iodizing is low, and it is commonly consumed by most people in fairly equal amounts (Nations System Standing Committee on Nutrition, n.d.). All large‐ and small‐scale salt manufacturers are required to be encouraged to fortify iodine adequately at the time of production. Firm quality assurance is important for them to follow. Moreover, consumers also need to develop awareness about salt usage (Charlton et al., 2012). Because of the active programs of salt fortification, IDDs are rapidly declining worldwide (Pandav, Yadav, Srivastava, Pandav, & Karmarkar, 2013). Consumption of other iodine‐enriched cooked food, vegetables, and fruits in regular diets can also be capable means to prevent IDDs (Elliott, 1987). There are some other sources of iodine, which can also contribute to diminishing IDDs.

## 2 SOURCES OF IODINE

Early (approximately 3600 B.C.) Chinese medical writings reveal records of decreases in goiter size upon consuming burnt sea sponge (Miles, 1998). Until that time, iodine was on its way to being discovered. However, these types of remedies remained active and their use continued globally. Iodine was discovered incidentally in the early 19th century (Rosenfeld, 2000). Iodine has an atomic number of 53 and atomic mass of 126.90447 amu, and it is available in the upper crust of the Earth as a trace element (Boyde, Mccorkell, Taylor, Bomphrey, & Doube, 2014). The variable geographic distribution of iodine is due to the effects of flooding, glaciation, and leaching into soil over time (Leung, Braverman, & Pearce, 2012). For these reasons, iodine is found mostly in coastal areas, and correspondingly, the common sources of dietary iodine are seafoods. Some iodine‐rich foods are described below (Gastaldi, Muraca, Beltramo, & Poggi, 2014; Hamza, Hewedi, & Sallam, 2013; Zimmermann & Boelaert, 2015).

The ocean is the prime store of iodine‐rich foods; hiziki, kombu, kelp, arame, and wakame are common. Kelp contains the highest amount of iodine among any foods. One tablespoon of hiziki contains about 780 mcg, a 1‐inch piece of kombu contains about 1,400 mcg, one tablespoon of kelp contains about 2,000 mcg, one tablespoon of arame contains about 730 mcg, and one tablespoon of wakame contains about 80 mcg of iodine. Different fruits are other great sources of iodine and cranberries are among them. Almost one ounce of cranberries contains approximately 100 mcg of iodine. Strawberries are very tasty and widely chosen for their attractive flavor. Approximately 13 mcg of iodine is found in a cup of fresh strawberries. Different beans are good dietary sources of iodine. Potatoes are also rich sources of iodine among vegetables. One medium‐sized baked potato contains 60 mcg of iodine.

Nascent iodine is a consumable form of iodine, and it contains an electromagnetic charge. It is like the precursor form of iodine which converts into THs. It permits greater release of energy once consumed. As a result, the human body can recognize and assimilate this form easily. Different commercial brands of this product are available. Lugol’s solution contains 85% distilled water, 10% potassium iodide, and 5% elemental iodine. Lugol’s solution increases respiratory tract secretions and prevents TH secretion. It is a widely administered commercial iodine source. Different solid dosage forms of potassium iodide are available in the form of tablets. These are closely bound inorganic forms of iodine and around 20% is assimilated into the body. They have been administered as emergency treatment for hyperthyroidism. They block the uptake of radioactive iodine in the thyroid gland and, therefore, lower the chances of thyroid cancer development. Besides, many other dietary iodine sources are available.

## 3 THE ROLE OF IODINE IN THYROID FUNCTION

Thyroid function is crucial to the metabolic functions of almost all tissues. The action of the thyroid originates from two different iodine‐containing hormones. They are T3 and T4 (Vanderpump, 2017). Iodine is the rate‐limiting element that is used to synthesize the THs. The thyroid gland is small; it is positioned at the front of the neck and weighs <1 ounce. It is found below the “Adam’s apple” or larynx. It has two halves (lobes) and lies along the windpipe (trachea; Can & Köksal, 2015). The lobes are linked to each other by a narrow group of thyroid tissues, which are known as the isthmus. The principal physiological role of iodine in the human body is to produce the THs. The association between iodine and thyroid function has been acknowledged since the early 20th century. Major forms of iodine are reduced to iodide in the body’s gut. Iodine is almost fully absorbed in the stomach and duodenum and is eliminated from the circulation largely by the thyroid gland and kidneys. In normal circumstances, the half‐life of plasma iodine is nearly 10 hr (Braverman, Cooper, Werner, & Ingbar, 2013). This is shortened when the thyroid gland is overactive. The mean turnover of iodine by the thyroid gland is approximately 60–95 μg in adults in iodine‐sufficient areas per day. A healthy adult usually retains 15–20 mg of iodine, 70–80% of which is in the thyroid gland. T3 and T4 degradation in the periphery releases iodine, which re‐enters the plasma iodine pool. The majority of this is excreted in urine, while very few amounts are present in feces. The mammary gland also has iodine‐related functions. It concentrates iodine and, later, secretes this into breastmilk that is provided to newborns. Small amounts of iodine are often found in the gastric mucosa, salivary glands, and choroid plexus (Spitzweg, Joba, Eisenmenger, & Heufelder, 1998).

The role of the thyroid gland is to convert iodine into the T3 and T4 THs. Cells of this gland are capable of absorbing iodine. These cells then combine iodine with the amino acid tyrosine and form T3 and T4, which are then released into the bloodstream. In fact, they move within the body where they control metabolism. All cells in the human body depend on T3 and T4 for the regulation of their metabolism. Iodide controls thyroid function by decreasing the response of the thyroid gland to thyrotropin (TSH) and preventing its own oxidation. Thus, it reduces its trapping after a delay. Minor alterations in iodine intake are adequate to rearrange the thyroid function system influencing serum TSH levels. Hence, iodide contributes to the negative feedback loop by modulating the thyroid gland’s response to TSH. With increased doses of iodide, its organification increases initially and later reduces. Iodide is known to inhibit numerous metabolic steps in thyroid cells in vitro (Guideline: Fortification of Food‐Grade Salt with Iodine for the Prevention and Control of Iodine Deficiency Disorders, 2014). It prevents the cyclic adenosine monophosphate cascade and also the Ca^2+^‐phosphatidylinositol 4,5‐bisphosphate (PIP_2_) cascade. Similarly, it activates H_2_O_2_ synthesis and, by this process, protein iodination in the thyroid gland (Massart et al., 2011); Corvilain, Laurent, Lecomte, Vansande, & Dumont, 1994).

## 4 COGNITIVE FUNCTION AND IODINE

**Figure 1:**
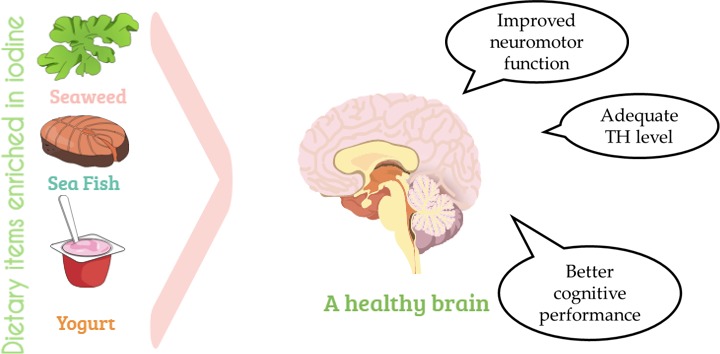
Iodine‐containing foods can contribute to healthy brain formation. TH, thyroid hormone

Iodine is found in the human body in an amount of 10–15 mg. Almost 70–80% of this iodine is available in the thyroid gland, and 90% of this is bound with thyroglobulin. Iodine confers many favorable health benefits. THs are able to bind with thyroid receptors in the nuclei of cells, and regulate gene expression within target tissues, such as the brain (Hetzel, Chavadej, & Potter, n.d.). Brain development is followed by different steps of maximal growth velocity (Dobbing & Sands, 1973; Girard, Koob, & Brunel, 2016). In fact, one important phase occurs during the first and second trimesters between the third and fifth months of gestation (Lignell et al., 2016). This includes neuronal multiplication and migration, as well as organization (Glinoer, 2001). Another phase occurs from the time of the third trimester to the second and third years postnatally. This phase includes glial cell multiplication and migration, as well as myelinization (Konin & Bhinder, 2013). Meanwhile, the first phase occurs earlier, when fetal thyroid reaches functional levels (Hetzel & Hay, 1979). In this phase, the adequate flow of THs to the developing fetus almost originates from the mother (Mann & Plant, 2010). However, during the second phase, smooth flow of THs to the fetus is essential, not only for fetal development. IDDs cause problems that are mostly irreversible. The results of ID during pregnancy, on pregnancy outcomes and early infant development, are very important. The initial impacts of ID in the fetus may be commenced by reduced maternal T4 transference before the onset of fetal thyroid function. In the first trimester of pregnancy, the fetus is reliant on maternal T4. Type II deiodinase is found from the maternal T4, and thus, the developing fetal brain with T3, which is the active hormone and binds to particular receptors. In actuality, the main action of fetal thyroid function becomes active in the second trimester (Morreale de Escobar, Jesús Obregón, & Escobar del Rey, 2000). During birth, 20–40% of T4 is available in the cord blood, which originates from the mother. This shows that thyroid profiles of newborns are affected by mothers’ iodine profiles. ID causes irreparable impairment in developing fetal brains. THs have important functions in various neurobiological processes. Dietary supplements enriched with iodine can play an important role that is presented in Figure 1. Deficiency of THs in the first and second trimesters could alter visual processing, attention, and visuospatial performance. However, during the third trimester, TH deficiency disturbs gross motor performance, memory, and motor activity. Deficits in THs could disturb language and verbal development, along with attention and memory skills postnatally (Schroeder & Privalsky, 2014).

Developmental neuropathologic and epidemiologic data revealed that from almost 12–14 weeks to 20–30 weeks of gestation could be the most important period wherein damage may occur (Jianqun et al., 1986). During 12–18 weeks, striatal and cortical neuronal proliferation, migration, and early formation of neuropil occur (Herschkowitz, 1988). During the same time, cochlear development also occurs. These data showed that, to avoid cretinism, iodine repletion should occur within the first three months of pregnancy. ID can cause reduced brain weight, and a greater mass of cells at the cerebral cortex and lower mass of cells in the cerebellum. Thus, neurological cretinism mainly initiates from maternal hypothyroidism due to ID. More evidence has shown that even moderate or mild hypothyroxinemia at the time of pregnancy potentiates the risk of neurodevelopmental deficits in offspring (Vermiglio et al., 2004). Alterations in the psychomotor growth of children affected by ID becomes noticeable later, at the age of about 2.5 years (Morreale de Escobar & de Vijlder, 2003). Within the brutal ID affected areas, children with lower T4 levels have lower performance compared with that in children with normal T4 levels (Glorieux, Desjardins, Letarte, Morissette, & Dussault, 1988). This is revealed in the results of reading, spelling, and cognitive performance tests (Huda, Grantham‐McGregor, Rahman, & Tomkins, 1999). Low T4 levels show different problems like neurological changes, weak memory function, and poor learning performance in school based on different validated tests of psychomotor function (Hoddinott et al., 2013). Different observational studies have shown that ID is associated with mental performance. Even children living in ID areas, but born in iodine‐sufficient areas, showed better cognitive performance than children born and living in ID areas (Qian et al., 2005). In a comparison of learning ability and motivation in severe and mild ID populations, it has been revealed that children with severe ID are slow learners. Moreover, children with severe ID have poor intelligence quotients compared with those in children with mild ID (Aboud, Bougma, Lemma, & Marquis, 2017; Tiwari, Godbole, Chattopadhyay, Mandal, & Mithal, 1996).

## 5 IODINE IN REGULAR FOOD SUPPLEMENTATION

There are recommendations for iodine and other nutrients in the dietary reference intakes (DRIs), which are provided by the Food and Nutrition Board (FNB). The DRIs are a collection of reference values that are accountable for planning and evaluating nutrient intakes in the healthy population. These values vary with gender and age. The following terminologies are used to describe the DRI (“Iodine—Health Professional Fact Sheet, 2018”).

**Figure 2:**
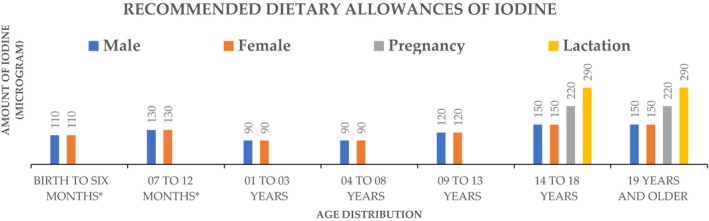
Recommended dietary allowances of iodine. *Adequate Intake

**Figure 3:**
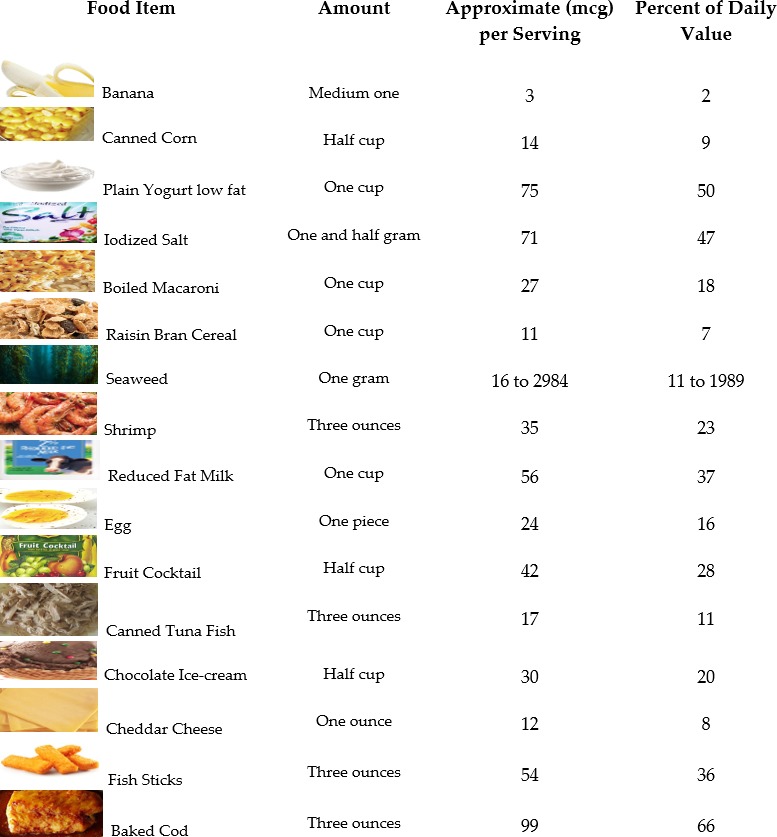
Iodine‐containing food sources with their values

Recommended dietary allowance (RDA): This is the daily average amount that is sufficient to fulfill the requirements of nutrients in almost all (97–98%) healthy people. Adequate intake (AI): This is a value used when intake is inadequate to fulfill the RDA. It also set at a level considered to confirm nutritional suitability. Estimated average requirement (EAR): This is the average, regular amount that is also considered and estimated to fulfill the necessities of 50% of healthy people. Based on age and sex, Figure 2 presents the iodine requirements for each day (“Dietary Reference Intakes for Vitamin A, Vitamin K, Arsenic, Boron, Chromium, Copper, Iodine, Iron, Manganese, Molybdenum, Nickel, Silicon, Vanadium, and Zinc, 2001”). However, these values can vary according to geographical circumstances. In our regular diet, we avail some foods on a regular basis. These are presented in Figure 3 with their iodine values (25 Iodine Rich Foods You Should Include In Your Diet, 2014; Dasgupta, Liu, & Dyke, 2008; Joordens, Kuipers, Wanink, & Muskiet, 2014; Pennington et al., 1995; Teas, Pino, Critchley, & Braverman, 2004).

## 6 PREVALENCE OF IODINE DEFICIENCY AND DISORDERS

### 6.1 Iodine deficiency during pregnancy

ID in pregnancy causes disturbances in THs in both mothers and fetuses. Lack of TH supply to the developing brain may cause cognitive impairments (Mehran et al., 2017). As THs function to ensure normal growth and development, consequences of TH disturbances are noticeable on the proportion of cell differentiation and gene expression (del Valdés Hernández et al., 2017). In fetal and primary postnatal life, different specific genes are expressed in brain regions due to the exerted TH action through the binding of T3 to nuclear receptors (Bernal, 2000). Binding of T3 with nuclear receptors is mainly dependent on the indigenous intracellular production from T4, which arises through type II deiodinase (Guadaño‐Ferraz, Escámez, Rausell, & Bernal, 1999). The crucial unwanted effect of ID is harm to the fetus. Treatment of ID through the regular diet in pregnant women decreases fetal and perinatal mortality, as well as progresses motor and cognitive function in the offspring in deficit areas (Trofimiuk‐Mudlner & Hubalewska‐Dydejczyk, 2017). Utero ID causes disorders followed by gross mental obstruction and cretinism (Lavin, 2009; Zimmermann, 2009).

### 6.2 Neonatal iodine deficiency

It takes time for the brain to develop to its full size. At the time of birth, the brain is only about one‐third of its full size. It proceeds to grow until the second year of life (Dobbing, 1974). Different studies have revealed that THs help supply iodine, which is important for normal brain development. The continuing presence of ID affects neonatal thyroid function, which is a risk to early brain development (Delange et al., 1986). A greater risk of poor mental growth in people who have severe ID is later shown through the existence of cretinism. ID is also important in the neonatal period due to increased susceptibility of the thyroid gland to radioactivity (Taylor et al., 2017). Uptake of radioiodine through the thyroid gland reaches its extreme values in the initial years of life. After that, the uptake declines progressively into adult life. Turnover rates of thyroidal iodine are higher in young infants than in adults; they reduce gradually with increasing age. To maintain normal TH levels, a greater rise in turnover rates is necessary in those with ID. Therefore, neonates and fetuses are significantly susceptible to ID. In addition, ID causes increased uptake of radioiodide following nuclear radiation. By overcoming ID, this increased uptake can be inhibited.

### 6.3 Iodine deficiency in childhood

In ID, it has been demonstrated that fine motor skills, information processing, and visual problem‐solving are markedly enhanced in school‐attending children following iodine administration. Scientific analysis suggested that ID in mild to moderate levels might prevent children from achieving their full intellectual ability. To some extent, the intelligence quotient frequency distribution in seemingly normal children is shifted toward lower values. This is related to children who did not experience ID in utero because of the correction of deficiency in their mothers before or at the time of initial gestation, which is when ID is most severe.

### 6.4 Iodine deficiency in adulthood

In adults, iodine acts as a significant determinant of thyroid disorders. Hypothyroidism and goiters due to the increase in thyroid activity cause severe ID (Zimmermann, 2016). In mild to moderate ID, increased thyroid action can compensate for minimal iodine consumption, causing the persistence of euthyroidism in a maximum number of people. Nonetheless, long‐lasting thyroid stimulation causes higher prevalence of these two diseases. This high prevalence of nodular autonomy usually results in a further increase in the prevalence of hyperthyroidism if iodine intake is subsequently increased by salt iodization. Yet, this increase is temporary as iodine adequacy eases thyroid activity.

## 7 SCENARIO OF IODINE IN HEALTH ECONOMICS

To make feasible, a general financial plan for ensuring adequate iodine consumption in the entire population is too difficult. Instead, efforts to measure the costs of ID could be a matter of weak assumptions and many mistakes may occur. Financial plans could be based on the geographical distribution; for example, the expenses in rural areas would not be equivalent to those in urban or geographical areas. Likewise, expenses in first‐world countries would not be equivalent to those in third‐world countries. Accounting the expenses, excluding the related benefits, could be a meaningless initiative comparatively. One initiative is iodization of all salt for human and animal consumption, which is known as USI. This is the safest, simplest, and cheapest initiative to ensure acceptable iodine consumption. A report from the World Bank suggested that this initiative may cost around USD 0.05 for each child every year “WHO | Micronutrient deficiencies” (2013).

### 7.1 Circumstances

There would be a responsibility of top international agencies like the United Nations Children’s Fund, World Health Organization, Canadian International Development Agency, United Nations Development Programme, Swedish International Development Cooperation Agency, United States Agency for International Development, World Bank, and other foreign support initiatives of other developed countries to ensure that the essential capital is available and to manage the required distribution to lessen ID and IDDs. To execute the program and project, a true backing should be available for the desired administrative expenses. For the initiative to be successful, different countries should also actively contribute.

### 7.2 Evaluation

Very few studies have been conducted about the expenses of preventative programs for IDDs. Further investigations regarding the expenses are required (Pandav, 2012). Expenses of evaluation would rely on the scale of initiatives, including the preferred tools, respective staff, as well as the total work involved (Völzke et al., 2016). However, specifically in cases of cretinism, the costs would be minimal as the information is only for a specific IDD, rather than for all IDDs. To evaluate all IDDs, the expenses would be large. Nonetheless, the extent of this evaluation would follow mostly on practical, political, and local situations. Further study and attention are required. The benefits of these evaluations that would lead to interferences to prevent or eliminate IDDs are immense.

### 7.3 Advantages

Studies on advantages should consider the expenses required for IDDs, although it is very difficult to account them all. Numerous efforts must be considered to measure the advantages of actions taken to prevent IDDs (Deficiencies, Howson, Kennedy, & Horwitz, 1998). Nevertheless, a tried and experienced way is available, which involves fortifying a widely available and commonly used ingredient: salt. There is a sizeable international body, which is working to establish a malnutrition‐free world, known as the Global Alliance for Improved Nutrition (GAIN; Hemery et al., 2018). GAIN is working on USI in 17 countries. This organization is working with national initiatives to acquire adequate iodine nutrition in different countries, including China, Egypt, Bangladesh, Ethiopia, India, Indonesia, Ghana, Niger, the Philippines, Russia, Pakistan, Senegal, Ukraine, and Nigeria. More organizations should be included to cover all countries worldwide.

## 8 FORTIFICATION STRATEGY

Food fortification is a process that helps billions of people worldwide to lead a life free from physical and mental impairments. Salt fortification is a similar process. In some countries, limited access to various food supplies, food shortages, and shortages in marketplaces or even in the quantity of iodine in the soil that vegetables and crops grow in leading to ID among the population is at concerning levels. The long‐term impact of this is negative, and global studies suggest that on average, iodine‐deficient communities suffer a loss of about 13 intelligence quotient points in the population. USI is the most convenient means to introduce an effective solution. USI requires some upfront investment, but offers a cheap and effective means to prevent ID. Country‐specific standards on iodization levels must be updated for the understanding of salt levels consumed in the diet to improve. Local producers in some countries do not possess the tools or financial incentives to adequately iodize salt. Different international organizations are working on filling this gap. These organizations are introducing more efficient models of salt iodization using sustainable partnership models and, where possible, market‐based approaches. There is an opportunity to proceed even further and eliminate ID globally through targeted programs in iodine‐deficient populations. As an example, in Ghana, the supply system established by GAIN has produced 1,750 metric tons of salt, ensuring that almost 6 million of their people have access to iodized salt every month (Global Alliance for Improved Nutrition, 2014). In history, this has been one of the most prominent illustrations of micronutrient malnutrition, which still affects hundreds of millions of people worldwide. In Ethiopia, in 2014, the initiation of a program was planned for 14 metric tons of iodine to cover 55 million people. They settled and applied a management information system for the government to progress the monitoring of iodization and quality of the nationwide iodized salt supply system in India. This approach takes the form of a state‐of‐the‐art, web‐based management system to track the production and movement of iodized salt. Regarding USI, the progress thus far has been remarkable. There was virtual eradication of IDDs in much of Europe and North America due to salt iodization. In the interim, among developing countries, iodine nutrition has improved drastically through effective salt iodization. As a result, the number of iodine‐deficient countries decreased from 54 in 2003 to 32 in 2011 (Andersson, Karumbunathan, & Zimmermann, 2012). In fact, there remains much work to eliminate ID globally (de Benoist, McLean, Andersson, & Rogers, 2008). Many people are still suffering the consequences of ID. More programs like GAIN should be operated to eliminate ID and to also ensure appropriate cognitive development (ID is not the only the factor that affects cognitive function).

There are two different chemical forms of iodine available that are used for salt iodization: iodate and iodide (Hong et al., 2012). Usually, food‐grade sodium chloride is processed. A range of about 30–200 ppm is the level of iodine fortification process. An important aspect is that iodides are more susceptible to degrade upon contact with impurities. However, iodates are to some extent more stable (“Food Fortification Technology, 2017”). There are four major ways by which iodine is added to the salt. These are drip‐feed addition, dry‐mixing, spray‐mixing, and submersion (“Fortification of foods: Historical development and current practices, 2017”).

Any kind of variation in food composition show significant impact in terms of iodine intake among young children who receive a good amount of their regular nutrient consumption from milk (Bath et al., 2017; Stevenson, Drake, & Givens, 2018). Therefore, iodization of milk may also be an important way to overcome ID (Hennessy et al., 2017). Fortification of flour, sugar, bread, and condiments with iodine could also be hopeful means to contribute to ID minimization (Charlton, Probst, & Kiene, 2016). Moreover, in animal products, fortification could be beneficial to increase the iodine content. Using slow‐release resins with water, dietary intake of iodine can be increased (Food and Agriculture Organization of the United Nations., 1996). In processed foods, added iodized salt could be a source of iodine. Pickled products and cured fish are good examples.

The interaction between iodine and other dietary factors should be understood, as via interactions, iodine forms can change, reducing its benefits as a nutrient (Truong, Baron‐Dubourdieu, Rougier, & Guénel, 2010). To determine optimum iodine fortification procedures and to implement them, further studies are required.

## 9 CONSEQUENCES OF IODINE DEFICIENCY IN COGNITIVE PERFORMANCE AND ITS CORRECTION

Cognitive impairments are the main consequences of ID. One of them is endemic cretin. When ID is severe and persists for long durations, then this effect is observed. From the second trimester of pregnancy, the damage initiates and if iodine is supplied, then it reverses (Lazarus, 2000). However, after the end of the second trimester, the damage continues irreversibly and causes severe cognitive impairments, such as speech and hearing problems (Prado & Dewey, 2014). Typically, patients with cretins are manageable and they can also perform simple tasks. Lower risk levels of ID cause lower degrees of impairment. The quantity of people affected is more than the quantity of people facing the effects of severe deficiency. From the modest, this alteration extends and then noticeable neurological deviations may cause reduced learning levels and grades in school (Khattak, Khattak, Ittermann, & Völzke, 2017). By this way, ID hampers socioeconomic advancements among the population (Mtumwa, Ntwenya, Paul, Huang, & Vuai, 2017).

To improve the value of life, alleviation of ID contributes a vast role. It eliminates cretinism and other minor grades of neuromotor and cognitive activity impairments, and thus improves survival.

## 10 CONCLUSION AND PERSPECTIVE

A healthy diet is essential from many aspects to lead a healthy life. Inclusion of adequate iodine in the regular diet for human body regulation at different ages plays a significant role. Ensuring sufficient amounts of iodine for the global population community is obligatory for achieving perfect cognitive performance, although some other factors are involved. If various barriers, like the lack of awareness and economic challenges, could be overcome, then the benefits of dietary iodine will be exposed.

## CONFLICTS OF INTEREST

The authors declare that they have no conflicts of interest.

## ETHICAL APPROVAL

It is not applicable.
