# Corrigendum

**DOI:** 10.1002/fsn3.2123

**Published:** 2021-01-19

In the article by Choudhry and Nasrullah (2018) there is an error in the 6th line of the Abstract section. The correction is given below (in bold).

Iodine, a dynamic nutrient present in thyroid hormones, is responsible for regulating thyroid function, supporting a healthy metabolism, and aiding growth and development. Iodine is also essential for brain development during specific time windows influencing neurogenesis, neuronal and glial cell differentiation, myelination, neuronal migration, and synaptogenesis. About 1.5 billion people in 130 countries live in areas at risk of **iodine deficiencies (IDs)**.
